# Transcriptomic and physiological responses of contrasting maize genotypes to drought stress

**DOI:** 10.3389/fpls.2022.928897

**Published:** 2022-08-03

**Authors:** Yifan Wang, Haoxue Guo, Xi Wu, Jiarui Wang, Hongjie Li, Renhe Zhang

**Affiliations:** College of Agronomy, Northwest A&F University, Yangling, Shaanxi, China

**Keywords:** maize, drought stress, transcriptome sequencing, physiological analysis, tolerance mechanism

## Abstract

Drought is a significant environmental stress factor that adversely affects maize productivity. However, many details regarding the molecular mechanisms of maize against drought are still unclear. In this study, leaf transcriptomics and physiological traits of two maize genotypes with differing drought resistance were analyzed. Transcriptome sequencing identified 8985 and 7305 differentially expressed genes (DEGs) in SD902 and SD609, respectively. Functional analysis suggested that numerous genes are highly involved in oxidative defense, protein modification, photosynthesis, phytohormone response, MAPK signaling, and transcription factors (TFs). Compared to SD902, SD609 had a higher expression of DEGs related to antioxidant enzymes, photosynthetic electron transport, heat shock proteins, and indole-3-acetic acid (IAA) signaling under drought conditions, which might contribute to its tolerance mechanisms to drought. Stress-induced TFs may play a crucial regulatory role in genotypic differences. Moreover, the physiological changes and gene expression abundance determined using quantitative reverse transcription polymerase chain reaction were consistent with the RNA sequencing data. The study results suggest that the higher drought tolerance of SD609 than SD902 can be attributed to stronger stress defense capabilities, IAA signal transduction, and more stable photosynthesis. Our findings provide new insights into the molecular mechanisms of maize against drought stress, and the candidate genes identified may be used in breeding drought-tolerant maize cultivars.

## Introduction

Maize (*Zea mays* L.) is a versatile cereal crop grown worldwide due to its economic and nutritional value in industry, food, and animal husbandry. However, the growth and development of maize plants are highly susceptible to drought conditions that hamper both early seedling establishment and the entire growth cycle, ultimately reducing yield potential (Fahad et al., 2017). Recently, extreme climates have resulted in more frequent and severe weather events, such as drought (Liu et al., 2010; Leng and Hall, 2019). The increase in soil desiccation, together with the increasing world population, has put greater pressure on maize production (Ray et al., 2018). There is an urgent need to improve the drought tolerance of plants using genetic engineering to alleviate this crisis (Leng and Hall, 2019). Hence, it is extremely important to identify the molecular mechanisms regulating the drought stress response, which would be useful for cultivating tolerant crops and ensuring food production security.

Generally, drought results in an extensive range of changes in plants including stomata closure, inhibition of photosynthesis and metabolic reactions, and excessive reactive oxygen species (ROS) generation, thereby causing oxidative stress in the plant cells and affecting overall plant growth (Suzuki et al., 2012). To cope with drought stress, plants institute several alterations at physiological and molecular levels. To protect against the damaging effects of ROS, plants activate antioxidant enzymes and defense proteins, such as superoxide dismutase (SOD), catalase (CAT), peroxidase (POD), ascorbate peroxidase (APX), glutathione (GSH), and HSPs (Ramachandra Reddy et al., 2004). At the same time, drought responses involve a complex regulatory network that controls the expression of stress-responsive genes (Liu et al., 2021). For instance, plant hormones such as abscisic acid (ABA), auxin, and cytokinin modulate the transcription of many drought-inducible genes (Fahad et al., 2015). Transcription factors (TFs) including MYB, NAC, and bHLH can interact with *cis*-regulatory sequences to trigger gene transcription and translation, providing adaptation to water deficits (Liu et al., 2021). Thus, the identification of specific genes and pathways correlated with drought tolerance is a fundamental advancement in the improvement of drought-tolerant varieties.

Transcriptome sequencing has become an effective technology that provides huge amounts of gene information to comprehensively characterize molecular regulators and investigate the genes involved in the response to abiotic stresses (Kumar et al., 2015; Jia et al., 2020). Several attempts have been made to understand the molecular mechanisms of drought in rice (Jia et al., 2020), wheat (Dugasa et al., 2021), sorghum (Zhang et al., 2021), and mung bean (Kumar et al., 2020). Drought stress induces differentially expressed genes (DEGs) in maize, mainly including TFs, hormonal signaling, stress defense, detoxification, and photosynthesis-related genes (Liu et al., 2021; Waititu et al., 2021), which are valuable for revealing the molecular mechanisms by which maize responds to drought. These studies highlight that the molecular responses to stress are strongly dependent on plant genotypes (Dugasa et al., 2021; Waititu et al., 2021). Regrettably, more detailed studies on changes at the transcriptional level under drought stress in maize plants are not yet sufficient.

In a previous study, Shaandan609 (SD609) was identified as a drought-tolerant genotype, and Shaandan902 (SD902) was identified as a drought-sensitive genotype (Li et al., 2021). Here, we compared the transcriptome profiles of the SD609 and SD902 genotypes in response to drought stress. Our objectives were to (i) reveal the transcript changes in drought-treated maize leaves and (ii) explore the underlying genes contributing to drought-tolerant mechanisms. Our findings deepen the understanding of maize acclimation to drought stress and provide valuable insights into drought tolerance improvement in maize plants.

## Materials and methods

### Plant material and drought treatment

The experiments were conducted from June through July 2021 in a rainproof shed at the Maize Experimental Station of Northwest A&F University, Yangling (34°283′N, 108°067′E), China. The two maize hybrids, SD609 and SD902, were obtained from Shaanxi Dadi Seed Co., China. Seeds of SD609 and SD902 were directly sown into plastic pots (diameter, 30 cm; depth, 45 cm) filled with 16 kg of air-dried clay and 10 g of a compound fertilizer consisting of N (24%), P_2_O_5_ (6%), and K_2_O (10%). Soil moisture is expressed as the percentage of maximum pot capacity (Ogbaga et al., 2014). All plants were watered to 85% water for 4 weeks. At the six-leaf stage, the plants were divided into two watering treatment groups. In the control group, the plants were watered daily to maintain the soil water content (SWC) at 85%. In the experimental group, the plants were allowed to dry by withholding water until a certain soil moisture content was reached (50% SWC). After 7 days, the top third of the fully expanded leaves was collected, frozen rapidly in liquid nitrogen, and stored at −80°C for subsequent measurements.

### Measurement of chlorophyll, gas exchange parameters, and energy conversion efficiency

The chlorophyll content was measured using a SPAD meter (SPAD-502, Konica-Minolta, Japan). Gas exchange parameters including net photosynthetic rate (Pn), stomatal conductance (Gs), intercellular CO_2_ concentration (Ci), and transpiration rate (Tr) were determined between 10:00 a.m. and 11:00 a.m. on the fully expanded third leaf from the top using a portable photosynthesis system instrument (LI-6400XT; LI-COR Biosciences, Lincoln, OR, United States) at a light intensity of 1,200 μmol/(m^2^ s) based on the method (Dai et al., 2020). According to the methods described by Zhou et al. (2019), the quantum yields of photosystem I (PSI) and photosystem II (PSII) from third fully expanded leaves were measured and calculated after adaptation to the dark for 30 min using a pulse amplitude-modulated system (Dual-PAM-100, Heinz Walz, Germany). The measured fluorescence parameters of PSII were involved in the effective quantum yield [Y(II)], quantum yield of non-regulatory energy dissipation [Y(NO)], quantum yield of regulatory energy dissipation [Y(NPQ)], and electron transport rate [ETR(II)]. The measured fluorescence parameters of PSI were involved in the effective quantum yield [Y(I)], quantum yield of non-photochemical energy dissipation due to donor-side limitation [Y(ND)], quantum yield of non-photochemical energy dissipation due to accept or side limitation [Y(NA)], and electron transport rate [ETR(I)]. Each measurement was repeated three times.

### Determination of physiological characterization and enzyme activity

Plant tissues (0.1 g) were used to measure the content of H_2_O_2_, O_2_^–^, and MDA in the control and drought treatment groups using the MDA Kit (MDA-2-Y), H_2_O_2_ Kit (H_2_O_2_-2-Y), and O_2_^–^ Kit (SAQ-2-G), respectively. The enzyme activities of SOD, POD, CAT, APX, GSH, and GR were measured according to the manufacturer’s instructions (SOD-2-Y, POD-2-Y, CAT-2-Y, APX-2-W, GSH-2-W, and GR-2-W, respectively). All the kits were obtained from Suzhou Comin Biotechnology Co., Ltd. (Suzhou, China). The activity levels of photosynthetic enzymes, including pyruvate orthophosphate dikinases (PPDK), phosphoenolpyruvate carboxylase (PEPC), NADP-malate dehydrogenases (NADP-ME), and Rubisco, were measured using an enzyme-linked immunosorbent assay (ELISA) kit (JINGKANG, Shanghai, China) according to the manufacturer’s instructions.

### Transcriptome sequencing and data analyses

The total RNA of all samples was extracted using TRIzol reagent (Invitrogen, United States). RNA concentration and quality were measured using an Agilent 2100 Bioanalyzer (Agilent Technologies, Palo Alto, CA, United States). The prepared libraries were sequenced using the NovaSeq 6000 platform. Clean reads were obtained from the raw reads by removing the adaptor sequences, low-quality sequences, and reads containing poly-N using Trimmomatic software. The clean reads were aligned to the reference genome from maize (the genome file was GCF_000005005.2, NCBI) using HISAT2.^1^ Fragments per kilobase of exon model per million mapped reads (FPKM) were used to estimate the expressed values and transcript levels. DESeq2 was used to identify the DEGs. |log2FC| > 1 and *p* value < 0.05, were used to screen the DEGs. The biological functions of all DEGs were annotated based on Gene Ontology (GO) and Kyoto Encyclopedia of Genes and Genomes (KEGG) pathway enrichment analyses. The ggplot2 and pathview R packages were used for plotting.

### Quantitative reverse transcription polymerase chain reaction analysis

Total RNA was extracted from the leaf samples (0.1 g) and ground into liquid nitrogen, using an RNA extraction kit (TIANGEN, Beijing, China). Reverse transcription (20 μL) was performed according to the manufacturer’s instructions (Fast Quant RT Kit, TIANGEN). The primer sequences for RT-PCR were designed using Primer3Plus and are listed in Supplementary Table 4. qRT-PCR was performed using a CFX96 real-time PCR machine (Hercules, CA, United States) with SuperReal PreMix Color (SYBR Green) (TIANGEN). The two-step PCR process was performed as follows: pre-denaturation at 95°C for 15 min, 40 cycles of 95°C for 10 s, and 60°C for 32 s. *GAPDH* (gene ID: 542367) was used as a reference gene to quantify the expression levels of the target genes according to the 2^–ΔΔCT^ method (Dong et al., 2020). Quantitative analysis of each RNA sample was repeated at least three times.

### Statistical analysis

Analysis of variance of all data was performed using SPSS 25.0, and Duncan’s multiple range test was performed with *P* < 0.05, indicating a significant difference. All values are shown as the mean ± standard deviation (SD) of three biological replicates. SigmaPlot 14.0 was used to achieve data visualization.

## Results

### Morphological and physiological responses of SD902 and SD609 seedlings to drought stress

**FIGURE 1:**
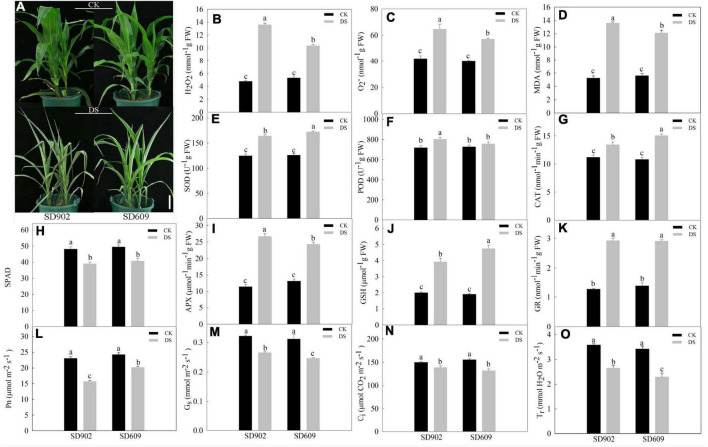
Effects of drought stress between the two maize genotypes. **(A)** Seedling phenotypic response of SD902 and SD609 during drought stress. Physiological effects of drought stress on reactive oxygen species (ROS) and MDA contents **(B–D)**, antioxidant enzyme activity **(E–G,I–K)**, chlorophyll concentration **(H)**, and gas exchange parameters **(L–O)**. The data shown are the means of three replicates (±SD) based on Duncan’s multiple range test. Means denoted with the same letter did not significantly differ at *P* < 0.05. DS, drought stress treatment; CK, well-watered treatment.

Seedlings of two contrasting varieties (SD902 and SD609) were exposed to natural drought stress treatment by withholding water for 7 days. Under well-watered conditions, no visible morphological differences were observed between the two maize genotypes. However, drought-induced stress resulted in leaf wilting and curling between the two genotypes of seedlings. Compared to drought-resistant SD609, SD902 exhibited more marked symptoms of drought sensitivity (extreme leaf wilting and curling) (Figure 1A). The physiological results showed that H_2_O_2_ and O_2_^–^ content markedly increased in both genotypes under drought conditions, but particularly in SD902 seedlings. The MDA levels were higher in SD902 than in SD609 (Figures 1B–D). Enzyme activity measurements showed that the activity levels of important antioxidants were differently increased in SD609 and SD902 under drought conditions. In brief, the activity levels of SOD, CAT, and GSH increased by 26.87, 28.26, and 59.76%, respectively, in SD609 and 23.93, 16.77, and 49.03% in SD902, respectively (Figures 1E–G). The activity levels of POD and APX in SD902 increased by 10.51 and 57.31% compared with those in SD609 by 3.92 and 46.08%, respectively (Figures 1I,J). The GR activity levels were not significantly different between the two genotypes (Figure 1K). Subsequently, drought significantly caused a decrease in the total chlorophyll concentration between SD609 and SD902 by the SPAD value, but the decrease did not cause significant differences between the two genotypes (Figure 1H). The gas exchange parameters suggested that SD609 had a higher net photosynthetic rate (Pn), followed by lower stomatal conductance (Gs), intercellular CO2 concentration (Ci), and transpiration rate (Tr) under drought conditions than SD902 (Figures 1L–O). These results indicate that SD609 might have good resistance performance under drought conditions.

### Transcriptome analysis of the two genotypes in response to drought stress

**FIGURE 2:**
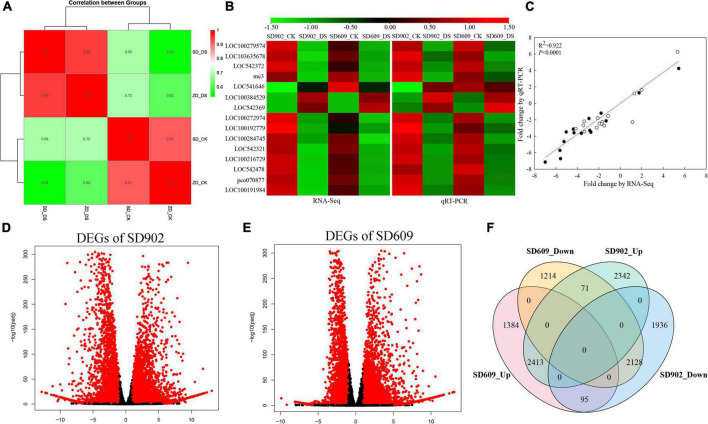
Genome-wide screen and expression analysis of differentially expressed genes (DEGs) between SD902 and SD609 based on RNA-seq. **(A)** Pearson correlation analysis of three biological replicates. **(B)** Validation of the RNA-seq results by qRT-PCR method. **(C)** Correlation analysis of gene expression value obtained from the RNA-seq and qRT-PCR. White and black circles, respectively, represent correlation of RNA-seq and qRT-PCR results in SD902 and SD609. **(D,E)** Upregulation and downregulation of DEGs between the two genotypes. Red and black dot symbols, respectively, represent significant difference genes and insignificant difference genes. **(F)** Venn diagram illustrating the number of upregulated and downregulated DEGs between SD902 and SD609.

To systematically understand the transcript changes in response to drought, the transcriptome profiles of leaf samples from SD609 and SD902 plants exposed to drought stress and well-watered treatments were analyzed using RNA sequencing. A total of 12 cDNA libraries were prepared from the collected samples and subjected to paired-end sequencing. The total number of raw reads in all libraries ranged from ∼22.71 to 39.12 million. After filtering out low-quality reads, clean reads were between 22 and 38 million, with 97.17% clean reads and 93.46% Q30 rate (Supplementary Table 1). Reads that could not be mapped to the maize genome were discarded, and only the mapped reads were further analyzed. Pearson correlation analysis suggested the credibility and repeatability of the RNA-seq data (Figure 2A). To verify the accuracy of the RNA-seq data, 15 mRNA transcripts were randomly selected, and their expression levels were analyzed using qRT-PCR (Figure 2B). The expression trends of the selected genes were consistent with the relevant transcripts. The changes in these genes assessed using qRT-PCR were similar (R^2^ = 0.922, *P* < 0.0001) to those determined using RNA-seq analysis, suggesting high reliability of the RNA-seq data (Figure 2C). Based on the standards of |fold changes| > 1 and *P*-value < 0.05, a total of 8,985 DEGs (4,826 upregulated and 4,159 downregulated) and 7,305 DEGs (3,892 upregulated and 3,413 downregulated) were screened in SD902 and SD609, respectively (Figures 2D,E). Overlapping analysis showed that 2,413 DEGs in SD609 and 2,128 DEGs in SD902 were upregulated and downregulated, respectively. Moreover, 2,342 and 1,936 DEGs were specifically upregulated and downregulated in SD902, respectively, while 1,384 and 1,214 in SD609, respectively (Figure 2F).

### Functional enrichment analysis of differentially expressed genes using the gene ontology and kyoto encyclopedia of genes and genome databases

**FIGURE 3:**
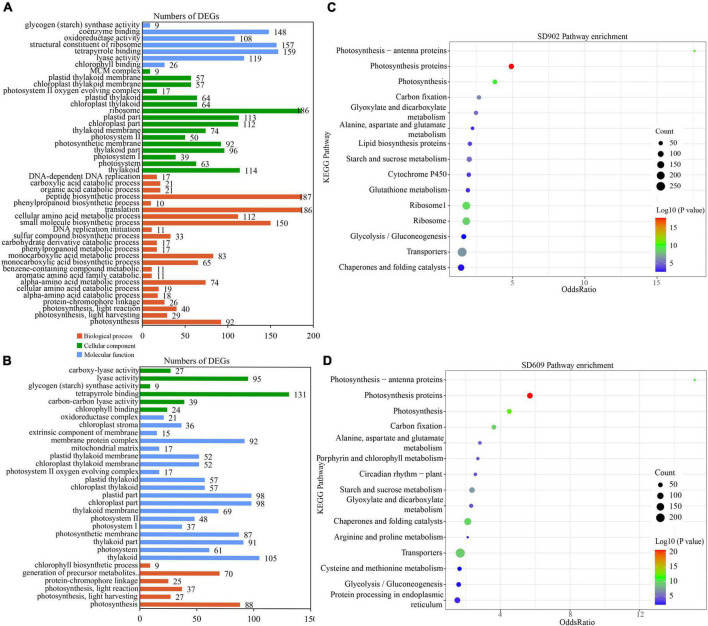
Gene Ontology (GO) and Kyoto Encyclopedia of Genes and Genome (KEGG) enrichment of differentially expressed genes (DEGs) between SD902 and SD609. **(A,B)** GO classification enrichment between the two genotypes. **(C,D)** Top 15 KEGG enrichment pathways of the two genotypes based on annotation information.

To determine the biological functions of DEGs between the two genotypes under drought conditions, GO and KEGG enrichment analyses were performed. In total, 55 GO terms were enriched in both SD609 and SD902, including 21 cellular components (CCs), nine molecular functions (MFs), and 25 biological processes (BPs) (Supplementary Table 2). In the two genotypes, the highly related CC terms were thylakoid (GO:0009579) and photosystem (GO:0009521). In the MF category, chlorophyll binding (GO:0016168) and tetrapyrrole binding (GO:0046906) were enriched after drought stress treatment. The most significant BP terms were photosynthesis (GO:0015979), light harvesting (GO:0009765), light reaction (GO:0019684), and protein–chromophore linkage (GO:0018298). Meanwhile, more BP terms [i.e., amino acid catabolic (GO:1901606, GO:0009063, and GO:0009074), benzene-containing compound metabolic (GO:0042537), monocarboxylic acid metabolism (GO:0072330 and GO:0032787), phenylpropanoid metabolism (GO:0009698), and carbohydrate derivative catabolism (GO:1901136)] were enriched only in SD902. A total of two BP terms, namely, the generation of precursor metabolites and energy (GO:0006091) and chlorophyll biosynthesis (GO:0015995), were enriched in SD609 (Figures 3A,B and Supplementary Table 2). Furthermore, KEGG analysis markedly enriched 77 pathways in the two genotypes under drought stress conditions (Supplementary Table 3). Interestingly, the highest enrichment pathway was photosynthetic proteins (ko00194) between SD609 and SD902. The top five enriched pathways were also related to photosynthetic reaction (ko00194, ko00195, and ko00196), carbon fixation (ko00710), starch and sucrose metabolism (ko00500), glutathione metabolism (Ko00799), and chaperones and folding catalysts (ko03110) (Figures 3C,D and Supplementary Table 3). These enriched pathways and related genes may be highly associated with drought tolerance regulation in both SD609 and SD902.

### Differentially expressed genes related to photosynthesis

**FIGURE 4:**
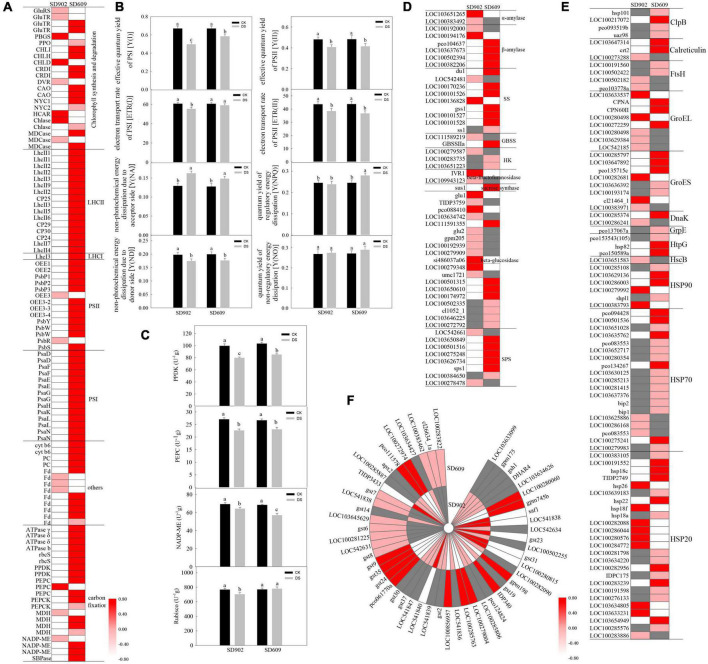
Responses of photosynthesis, energy conversion, protein protection, and AsA-GSH cycle between SD902 and SD609 under drought stress. **(A)** Expression profile of transcripts involved in photosynthesis. **(B)** Fluorescence parameters of PSII and PSd between the two genotypes by measuring photosynthetic efficiency. **(C)** Activity levels of key enzymes involved in carbon assimilation by ELISA analysis. **(D–F)** Expression profile of transcripts involved in energy conversion, protein protection, and AsA-GSH cycle. The heatmap is summarized by log2 (FC) of each gene from RNA-seq data. The data shown are the means of three replicates (±SD). Means denoted with the same letter did not significantly differ at *P* < 0.05. Y(I), effective quantum yield of PSI; Y(II), effective quantum yield of PSII; ETR(I), electron transport rate of PSI; ETR(II), electron transport rate of PSII; Y(NA), quantum yield of non-photochemical energy dissipation due to the acceptor side; Y(ND), quantum yield of non-photochemical energy dissipation due to PSI donor side; Y(NPQ), quantum yield of regulatory energy dissipation of PSII; Y(NO), quantum yield of non-regulatory energy dissipation of PSII; PEPC, phosphoenolpyruvate carboxylase; Rubisco, ribulose-bisphosphate carboxylase small chains; PPDK, pyruvate orthophosphate dikinases; NADP-ME, NADP-malate dehydrogenases; DS, drought stress treatment; CK, well-watered treatment.

Photosynthesis is sensitive to drought conditions in most plants. In the present study, numerous genes involved in chlorophyll metabolism, light energy transfer, and carbon fixation were significantly altered in both SD902 and SD609 after drought treatment (Figure 4). Among them, 22 chlorophyll biosynthesis and degradation-related genes were identified between SD902 and SD609 under drought conditions, including *glutamyl tRNA reductase* (*GluTR*), *porphobilinogen synthase* (*PBGS*), *protoporphyrinogen oxidase* (*PPO*), *Mg chelatase subunit I/H/D* (*CHLI/H/D*), *Mg-protoporphyrin IX monomethyl ester* (*oxidative*) *cyclase* (*CRDI*), *divinyl chlorophyllide a 8-vinyl-reductase* (*DVR*), *chlorophyllide a oxygenase* (*CAO*), *chlorophyll b reductase*, *chlorophyllase* (*Chlase*), and *Mg dechelatase* (Figure 4A). Compared to SD609, most of the genes were downregulated in SD902. Moreover, 17 genes concentrated in the light-harvesting antenna complex I (LHCI) and light-harvesting antenna complex II (LHCII) in the two genotypes were significantly downregulated after drought treatment (Figure 4A). In addition to the LHCI-related gene (*LhcI3*), other 16 DEGs were derived from the LHCII system, including *CP30*, *CP29*, *CP25*, and *CP24*. The expression levels of *LhcI3* between SD902 and SD609 decreased by 4.61 and 2.41 times, respectively. The expression levels of other LHCII-related genes decreased in SD902 by 1.83–11.16 times, and those of SD609 decreased by 1.67–4.55 times. By contrast, these genes displayed a weaker decrease in SD902 than in SD609 under drought stress.

A total of 41 genes correlated with photosynthetic electron transfer were identified in the two genotypes subjected to drought stress based on RNA-seq data (Figure 4A). In SD902, the expression of eight genes (*LOC100272890*, *LOC100191684*, *LOC107648855*, *LOC100281199*, *LOC100273117*, *LOC103627333*, *PSBQ1*, and *LOC103647735*) related to oxygen-evolving enhancer proteins were downregulated by 1.74–7.81 times, while those in SD609 were decreased by 1.68–3.33 times. The expression levels of five photosystem II reaction center-related genes (*PsbY*, *PsbR*, *PsbS*, and *PsbW*s) were significantly decreased in SD902 by 1.95–4.15 times, and their expression was only decreased in SD609 by 1.26–3.29 times. Compared with those in SD609, the expression levels of 14 photosystem I reaction center-related genes (*PsaD*, *PsaF*, *PsaE*, *PsaG*, *PsaL*, *PsaN*, *PsaH*, and *PsaK*) in SD902 showed a weaker decrease (by 2.83–6.10 times). Furthermore, the expression levels of the *Cyt b6/f complex* (*petB*), *plastocyanin* (*PC*), and *ferredoxin* (*Fd*) genes were affected by drought. Accordingly, 80 and 67% of these genes exhibited drastically decreased expression in SD902 (by 1.79–5.04 times) and SD609 (by 1.19–2.50 times), respectively. We also surveyed the energy conversion efficiency of the two maize genotypes under drought conditions (Figure 4B). Compared with drought-sensitive SD902, the SD609 genotype had a higher Y(I) (effective quantum yield), ETR(I) (electron transport rate), Y(NPQ) (the quantum yield of regulatory energy dissipation), and Y(NO) (the quantum yield of non-regulatory energy dissipation). Y(II) (effective quantum yield) and ETR(II) (electron transport rate) displayed slight differences in both SD902 and SD609. The Y(NA) level was higher in SD902 than in SD609. Y(ND) was significantly decreased in the two genotypes.

Upon drought stress, the expression levels of four genes encoding ATPase γ, ATPase δ, and ATPase b were decreased in SD902 (by 2.63–3.08 times) and SD609 (by 1.49–2.28 times), respectively (Figure 4A). The expression levels of 13 genes involved in CO_2_ assimilation were also significantly downregulated in SD902 by 1.29–5.57 times, whereas their expression was only decreased in SD609 by 1.17–2.71 times, such as *PEPC*, *sedoheptulose-1,7-bisphosphatase* (*SBPase*), *ribulose bisphosphate carboxylase small chains (rbcS*), *PPDK*, *phosphoenolpyruvate carboxykinases* (*PEPCK*), *malate dehydrogenases* (*MDH*), and *NADP-malate dehydrogenases* (*NADP-ME*) (Figure 4A). Accordingly, we measured the enzymatic activities of PEPC, PPDK, NADP-ME, and Rubisco using ELISA. The results showed that drought reduced PPDK and NADP-ME activities, causing significant differences between the two genotypes (Figure 4C). Although PEPC enzyme activity was significantly reduced compared with that under well-watered conditions, there was no marked difference between the two genotypes. Interestingly, Rubisco activity was downregulated in SD902 but upregulated in SD609 under drought conditions. In addition, some glucose metabolism-relate genes of the two genotypes were also changed during the drought condition (Figure 4D). More than 64 and 83% genes related to biosynthesis of starch and sucrose were downregulated in SD902 and SD609, such as *starch synthase* (*SS*), *granule-bound starch synthase* (*GBSS*), *hexokinase* (*HK*), *sucrose-6-phosphatase*, and *sucrose-phosphate synthase* (*SPS*). More than 47 and 67% of cellulose degradation-related genes exhibited significantly downregulated expression between SD902 and SD609, respectively. Beta-amylase and sucrose synthesis-related genes also changed differently in the two genotypes.

### Differentially expressed genes related to HSPs and antioxidants

HSPs are often induced by multiple stressors to protect proteins. According to our RNA-seq data, a total of 84 HSP encoding genes were changed between SD902 and SD609 under drought conditions, including *ATP-dependent Clp-protease ATP-binding subunits* (*ClpB*), *calreticulin*, *FtsH*, *GroEL*, *GroES*, *DnaK*, *GrpE*, *HtpG*, *HscB*, *HSP20*, *HSP70*, and *HSP90* (Figure 4E). The number of HSP genes was similar between SD902 and SD609, but their type and expression levels displayed obvious differences between the two genotypes. Compared to that in SD902, more HSP-related genes were upregulated in SD609, such as *ClpB*s, *FtsH*s, *HSP70*s, and *HSP20*s. The common *HSP*s had a higher upregulation in SD609 than in SD902. Moreover, we identified antioxidant defense-related genes in the two genotypes during drought stress (Figure 4F). Among them, the expression of four SOD-related genes (*LOC541646*, *LOC103626390*, *LOC100384855*, and *LOC100136885*) was decreased in SD902 by 1.32–1.74 times, whereas the expression of *LOC541646* and *LOC542722* genes was significantly increased in SD609 by 1.63 and 1.78 times. In total, three POD-related genes (*LOC100384529*, *LOC100194355*, and *LOC103504713* and two CAT-related genes (*LOC542369* and *LOC542230*) were differentially expressed in SD902 and SD609. Obviously, 49 GSH-AsA metabolism-related genes were changed under drought, including *glutamate cysteine ligase*, *glutathione dehydrogenase/transferase*, *glutathione peroxidase* (*GSH-Px*), *NADPH-glutathione reductase*, *glutathione S-transferase* (*GST*), and *L-ascorbate peroxidase* (*APX*). In SD902, the expression of 17 GST-related genes was increased by 1.10–3.90 time, while 10 GST genes were upregulated in SD609 by 1.07–2.16 times. The expression of APX-related genes was lower in SD902 cells than in SD609 cells. In general, these stress defense genes exhibited higher upregulation in SD609 than in SD902.

### Differentially expressed genes related to phytohormone and MAPK signal transduction

**FIGURE 5:**
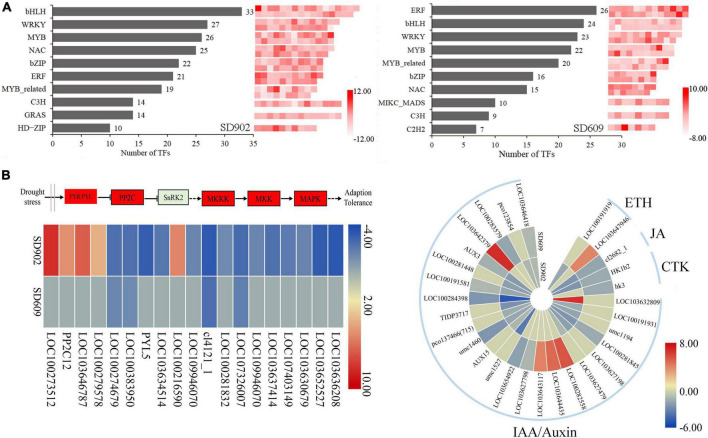
Expression levels of differentially expressed genes (DEGs) involved in transcription factors, MAPK, and plant hormone signaling between SD902 and SD609 based on log2 (FC) value. **(A)** Top 10 transcription factor families and the number of corresponding members. **(B)** Signal transduction of MAPK and plant hormone-related genes during drought conditions.

When subjected to drought stress, the expression of numerous genes related to plant hormone signaling was differentially altered in SD902 and SD609, including abscisic acid (ABA), ethylene (ETH), jasmonic acid (JA), cytokinin (CTK), and indole-3-acetic acid (IAA) (Figure 5B). Overall, nine DEGs in SD902 (five upregulated and four downregulated) and two DEGs in SD609 (downregulated) were annotated into the ABA pathway. Interestingly, the expression of four PP2C-related genes in SD902 was only upregulated by 4.20–8.26 times, while only *PYR/PYL* genes were downregulated in SD609. Moreover, 17 and 13 DEGs were annotated to the auxin-signaling pathway in SD902 and SD609, respectively. The expression of these genes was higher in SD609 than in SD902. Drought also affected the expression of other hormone signaling-related genes, including JA, CTK, and ETH. Among them, an ethylene-insensitive factor (*LOC103647946*) was only increased in SD609 by 4.04 times, while an ethylene-responsive transcription factor (*LOC100191919*) was only decreased in SD902 by 1.63 times. The expression of jasmonic synthetase (*cl2682_1*) and two CTK receptor (*HK1b2* and *hk3*) genes in the two genotypes was downregulated to varying degrees. Drought resulted in changes in the expression of genes involved in MAPK signaling (Figure 5B). A total of nine MAPK cascade-related genes (*MAPK1/8*, *MKK4/9*, and *MKKK17*s) were significantly decreased in SD902 by 1.42–3.44 times. However, compared to SD902, only two *MAPK*s (*MAPK8* and *MAKK9*) were decreased in SD609 by 2.48 and 1.85 times, respectively. These results suggest that the two genotypes may have different levels of regulation of plant hormones and MAPK signaling in response to drought stress.

### Differentially expressed genes related to transcription factors

Transcription factors play an essential role in regulating the expression of stress-responsive genes in plants (Wang et al., 2020). Our results identified 346 (154 upregulated and 192 downregulated genes in 39 diverse families) and 279 TFs (126 upregulated and 171 downregulated in 44 diverse families) in SD902 and SD609, respectively (Figure 5A). Many of these TFs were related to bHLH, WRKY, ERF, MYB, NAC, and bZIP proteins, suggesting that these TFs may participate in drought regulation in both SD902 and SD609 under drought conditions. In the two genotypes, the expression of TF *MYB166* and *WRKY29* was commonly upregulated, whereas that of *WRKY96*, *MYBR115*, *bZIP17*, *MYBR95*, *EREB68*, and *bHLH36* was commonly downregulated. However, the expression of some TFs [i.e., *bHLH104* (9.60-fold), *EREB34* (4.14-fold), *HDZIV14* (6.10-fold), and *MYB64* (−6.15-fold)] was altered only in SD902, whereas that of *EREB179* (6.84-fold), *bZIP111* (5.60-fold), *EREB27* (−4.06-fold), and *MYB103* (−3.72-fold) was altered only in SD609. Differences in the expression of these TFs might be vital for diverse drought resistance of the two genotypes.

## Discussion

Drought stress induces various effects on the photosynthetic system, ROS metabolism, stress defense, TFs, hormone response, and MAPK signaling in plants (Fahad et al., 2017; Dugasa et al., 2021). In this study, differences in gene expression profiles between the two maize genotypes (SD902 and SD609) under drought conditions were detected using RNA-seq. A total of 8,985 (SD902) and 7,305 (SD609) DEGs were detected in the two varieties. Functional analysis indicated that the pathways involved in photosynthesis, starch and sucrose metabolism, glutathione metabolism, chaperones and folding catalysts, TF regulation, plant hormone, and MAPK signal transduction were highly enriched in both genotypes after drought stress. Notably, the expression levels of the genes related to these enriched pathways were significantly different between SD902 and SD609. Likewise, we observed physiological changes consistent with gene expression between SD902 and SD609 under drought conditions. Accordingly, there appeared to be distinct degrees of drought regulation in the SD902 and SD609 genotypes with different levels of drought tolerance.

One of the vital physiological phenomena affected by drought in plants is photosynthesis (Al-Yasi et al., 2020). In this study, 84 genes related to photosynthesis were significantly different between SD902 and SD609 during drought conditions (Figures 4A–C). In detail, 13 chlorophyll synthesis-related genes (GluTR, PBGS, PPO, CHL/H/D, CRDI, DVR, and CAO) were significantly downregulated in the two genotypes. For example, GluTRs are vital rate-limiting enzymes in tetrapyrroles that control the chlorophyll synthesis rate (Wang et al., 2018). Interestingly, although the expression levels of chlorophyll synthesis-related genes were different in the two drought-treated genotypes, there was no significant difference in the chlorophyll content between SD902 and SD609. This result may be related to post-transcriptional modification of genes (Guerra et al., 2015), which suggests that chlorophyll synthesis in the two phenotypes may be similarly regulated at the protein level. PSII and PSI are mainly responsible for light energy harvesting and transfer, and the stability of PSII and PSI systems is closely related to photosynthetic efficiency (Borisova-Mubarakshina et al., 2020). Studies have suggested that a decrease in PSII and PSI system subunit-encoding genes can inhibit the photoreaction process (Guo et al., 2020). Drought treatment resulted in marked downregulation of chlorophyll a-b binding and electron transfer-related genes in SD609, especially in SD902. These decreased expression on genes may cause lower photosynthetic performance in drought-treated SD902 plants than in SD609 plants (Figure 1). Similar results were observed by Liu et al. (2021). In addition, we investigated the fluorescence parameters of the two genotypes under drought conditions to explore the possible tolerance regulation beyond the effects of gene regulation. Compared with drought-sensitive SD902, we observed higher ETR(I) and Y(I) in drought-resistant SD609, which might have resulted from a weaker decrease in PSI-related genes in SD609. Notably, SD609 had a higher Y(NPQ) level, which reflected a stronger energy dissipation ability. Energy dissipation is an important strategy for plants to reduce photoinhibition and protect the photosynthetic system (Ashraf and Harris, 2013). Although stomatal closure limits energy dissipation (Yokota et al., 2002), stomatal closure on SD609 leaves does not seem to significantly affect its heat dissipation, which may be an important regulation contributing to the photosynthetic stability of the drought-tolerant (SD609) genotype. The Calvin cycle is a key component of photosynthesis (Beyenbach and Wieczorek, 2006). Here, drought stress resulted in the downregulation of carbon assimilation-related genes in SD609, particularly in SD902. In addition to comparing gene expression, the activity levels of vital enzymes (PEPC, PPDK, NADP-ME, and Rubisco) were determined under drought conditions (Figure 4C). By contrast, PPDK and Rubisco showed high activity in drought-treated SD609. In C4 plants, these enzymes determine the utilization rate of CO_2_ in the intercellular space (Liu et al., 2017). The SD609 genotype may have higher CO_2_ utilization under drought conditions, which avoids excessive CO_2_ accumulation by metabolic reactions. In general, SD609 has a more stable photosynthetic system than SD902, which contributes to drought tolerance.

Under conditions of drought stress, ROS accumulation along with oxidized plasma membranes is a vital challenge for plant growth (Suzuki et al., 2012). To avoid the toxic effects of excessive ROS, plants have evolved detoxification mechanisms including antioxidant enzymes and antioxidant molecules (Anjum et al., 2017). Enzymatic ROS removal mainly involves SOD, POD, CAT, APX, and glutathione, which are essential for maintaining stable metabolism in plants. After drought stress, we observed a significant increase in the H_2_O_2_ and O_2_^–^ content between SD902 and SD609 (Figures 1B,C), suggesting that drought results in toxic effects in the two different maize cells lines. By contrast, the lower MDA level in drought-resistant SD609 could indicate a higher cell membrane stability index under drought stress conditions (Figure 1D). Therefore, drought-sensitive SD902 plants exhibited a more susceptible phenotype under drought conditions. In addition, similar changes have been observed in contrasting rice genotypes exposed to heat stress (Al-Zahrani et al., 2022). The DEGs involved in the ROS-removing system in SD902 and SD609 were detected after drought treatment, which were mainly annotated to SOD, POD, CAT, glutathione S-transferase, and L-ascorbate peroxidase (Figure 4F). This shows that the ROS-removing system may be activated when ROS accumulate excessively and produce toxic effects in the two genotypes (Qi et al., 2018; Li H. et al., 2019). The expression levels of genes encoding SOD, CAT, and GSH were higher in SD609 than in SD902, which may contribute to the high enzyme activity of SOD, CAT, and GSH, leading to a stronger ROS-removing capability. However, HSPs are responsible for protein assembly, folding, translocation, and degradation to stabilize proteins and membranes under growth and stress conditions (Haq and Shakeel, 2021). Here, 38 genes related to HSP70s and HSP20s were significantly upregulated in drought-treated SD609 relative to SD902 (20 upregulated), which might contribute to stronger protein repair ability to decrease drought-induced cell damage in SD609 (Figure 4E). Moreover, the expression levels of the genes encoding ClpB, calreticulin, FtsH, DnaK, GrpE, and HtpG were higher in SD609 than in SD902 plants. Previous research has shown that the expression of these *HSP*s plays a positive role in drought defense. For instance, calreticulin exhibits remarkable crosstalk with stress and phytohormone signals to modify proteins under stress environments (Joshi et al., 2019), and other molecular chaperones have been found to participate in the refolding of misfolded and aggregated proteins (de Marco et al., 2007). Overall, the higher expression of ROS-removing and HSP-related genes in SD609 may cause stronger detoxification and cell protection in leaves, which are consistent with its drought-resistant physiology and phenotypic traits under drought conditions.

Signal transmission is a crucial process for coping with stressful environments. Plant hormone signaling has multiple roles in affecting plant growth and recovering from stress damage (Ullah et al., 2018). This study identified five phytohormone signaling pathways in SD902 and SD609 plants subjected to drought stress (Figure 5B). The expression of ABA- and IAA-related genes showed obvious differences in quantity and type between the two varieties. Compared with that in SD609, many ABA signaling genes in SD902 were altered (i.e., all PYR/PYLs decreased and PP2Cs increased). PYR/PYL, an ABA signaling receptor, transmits ABA signaling together with the negative regulatory element PP2C (Zhao et al., 2020). Therefore, alterations in these genes may be related to the high ABA signal sensitivity of SD902. By contrast, more IAA signal-related genes in SD609 had higher expression, which may reduce the ABA effect owing to the antagonism of SD609. The effects on ABA and IAA signaling significantly affect plant phenotypic traits (Ding et al., 2015; Zong et al., 2020). Thus, the high expression of IAA genes in SD609 may be closely involved in the growth phenotype during drought stress. Moreover, as an important biological signal, the MAPK cascade is widely involved in plant stress responses (Moustafa et al., 2014). In this study, we identified three types of MAPKs (MAPK, MKK, and MKKK) after drought stress (Figure 5A). Compared to that in well-watered plants, the expression of these genes was decreased in drought-treated SD609, especially in SD902. Some MAPK protein-encoding genes negatively participate in maintaining ROS homeostasis and controlling numerous life events (Zhu et al., 2020). The expression differences in these MAPK genes in this study may be considered to cause various response signaling pathways in the two drought-resistant maize genotypes.

Transcription factors play key roles in activating the expression of downstream genes in response to environmental stress (Wang et al., 2020). The overexpression of ERF TFs in *Arabidopsis*, rice, tomato, and tobacco has been shown to enhance tolerance to diverse biotic and abiotic stresses (Zhang et al., 2010; Sharma et al., 2013). In this study, more than 60% ERFs were significantly upregulated in both genotypes, but the upregulation was much greater in SD609, for example, in ERFs such as ERF1, ERF34, and ERF65. These ERF TFs might play a regulatory role that contributes to the high drought resistance of SD609. WRKY TFs have been shown to participate in regulating transcription reprogramming under stress conditions (Chen et al., 2012). The results showed that more than 70% of WRKYs in SD609 were upregulated compared with that in the SD902 genotype, suggesting that transcriptional reprogramming events involving WRKY TFs may be more active in SD609 under drought conditions. NAC TFs effectively increase drought and heat stress resistance in rice by inducing stress-inducible genes (Hu et al., 2006). Here, 11 and 13 NACs were upregulated in both SD902 and SD609, respectively, indicating that these NACs might play an important role against drought stress in the two genotypes. Differences in gene number and expression abundance between SD902 and SD609 may be involved in drought performance under drought conditions. The expression of numerous bHLHs (more than 73%) was altered at the transcription level in SD902, especially in SD609, such as bHLH36, bHLH145, and bHLH104. The type of bHLH TFs has been shown to widely regulate stress adaptation (Li et al., 2016; Li Z. et al., 2019). For example, bHLH104 is involved in iron tolerance in plants (Yao et al., 2018), and MfbHLH38 effectively improves drought resistance in *Arabidopsis* (Qiu et al., 2020). These TFs may powerfully modulate drought response genes, and different alterations in their expression levels might play a vital role in the drought resistance of the two genotypes.

## Conclusion

**FIGURE 6:**
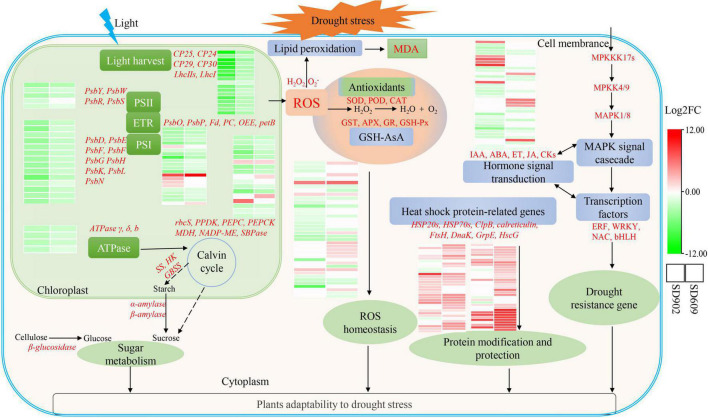
Working model for the regulatory mechanism of drought tolerance between the two maize genotypes under drought stress conditions. Heatmaps are summarized by log2 (FC) of mRNA levels. Red words represent vital DEGs identified in two maize genotypes.

In this study, a comprehensive comparison between drought-tolerant (SD609) and drought-sensitive (SD902) genotypes by integrating transcriptomics and physiological analysis provided a better understanding of drought tolerance mechanisms in maize. The physiological results demonstrated that SD609 was more tolerant to drought than SD902. RNA-seq data identified 8985 and 7305 DEGs in SD902 and SD609, respectively, which differentially affect the oxidative defense system, photosynthesis performance, and IAA signal transduction in the two genotypes. Moreover, drought also differentially changed the expression levels of TFs, which may explain the stronger drought resistance ability of SD609 than that of SD902. The co-expression of genes related to stress defense (*SOD*, *CAT*, *GSH*, *HSP70*, *HSP20*, and *ClpB*), photosynthesis (*PsbY*, *PsbW*, *PsaD*, *PsaE, PsaG*, *PsaH*, *PsaK*, and *petF*), and IAA signal (*LOC103642379*, *LOC100282558*, and *LOC103644435*) may be important for adaptation to drought in SD609. Accordingly, we present a working model for the regulatory mechanism of drought tolerance in SD609 (Figure 6), which can partially explain the high drought tolerance of SD609 and provide useful insights into the physiological and molecular mechanisms underlying drought tolerance in maize.

## Data availability statement

The datasets presented in this study can be found in online repositories. The names of the repository/repositories and accession number(s) can be found below: https://www.ncbi.nlm.nih.gov/, PRJNA765291.

## Author contributions

YW designed the experiment, analyzed the data, and wrote the manuscript. HG analyzed the data and wrote the manuscript. XW and JW carried out the experiment and provided some good suggestions on the manuscript. HL conducted a part of the experiment. RZ conceived the experiment, revised the manuscript, and funded. All authors have read and approved the manuscript.

## Footnotes

1
http://ccb.jhu.edu/software/hisat2/index.shtml
